# Empowering communities in combating river blindness and the role of NGOs: case studies from Cameroon, Mali, Nigeria, and Uganda

**DOI:** 10.1186/1478-4505-10-16

**Published:** 2012-05-10

**Authors:** Stefanie E O Meredith, Catherine Cross, Uche V Amazigo

**Affiliations:** 1 Global health Consulting, Divonne, France; 2 Independent Consultant, Haywards Heath, Lindfield, England; 3 Independent Consultant, Enugu, Nigeria

**Keywords:** Community–based, Community-directed, Onchocerciasis, Ivermectin distribution, NGO, APOC, MDP

## Abstract

The control of onchocerciasis is not only a major success story in global health, but also one of the best examples of the power of public-private partnership at the international level as well as at the national level. The onchocerciasis story is also a leading example of the contribution of a group of called Non-Governmental Development Organizations (NGDO) to operational research which resulted in important changes in treatment strategies and policies.

The four case studies presented here illustrate some key contributions the NGDOs made to the development of “community directed treatment with ivermectin” –CDTI, in Africa, which became the approved methodology within the African Programme for Onchocerciasis Control (APOC). The partnership between the international, multilateral, government institutions and the NGDO Coordination Group was the backbone of the APOC programme’s structure and facilitated progress and scale-up of treatment programmes. Contributions included piloting community–based methodology in Mali and Nigeria; research, collaboration and coordination on treatment strategies and policies, coalition building, capacity building of national health workforce and advocacy at the national and international level. While the Onchocerciasis Control Programme (OCP) and APOC provided leadership, the NGDOs working with the national health authorities played a major role in advocacy evolving the community methodology which led to achieving and maintaining- treatments with ivermectin for at least 20 years and strengthening community health systems.

## Introduction

Although the onchocerciasis story has been widely documented [1,2] and reviewed by e.g. the Center for Global Development [3] and Bush and Hopkins[4], it is important to highlight the strategies that have made it so effective. In this publication we focus on the role that NGDOs played in the early 1990s in empowering the communities affected by the disease in Africa to take charge of their own treatment programmes, which may be necessary to sustain for 20 years or more. In 1997 the resulting CDTI became the approved methodology within the African Programme for Onchocerciasis Control (APOC), and the community-directed strategy has since been further developed and adapted for use in many other health and development programmes [4].

This paper combines a review of published literature from a PubMed search using combinations of the terms “onchocerciasis”, “community based” , “NGO” and Loaisis, unpublished documents from Sightsavers archives the Non-Governmental Development Organization ( NGDO) Coordination Group for ivermectin distribution meeting reports, and structured interviews with 28 key stakeholders from national onchocerciasis programmes, NGDOs, OCP, APOC, Tropical Disease Research (TDR) and The World Bank.

### Onchocerciasis

Onchocerciasis, a vector-borne infection caused by the parasite *Onchocerca volvulus*, was highly endemic in many parts of Africa before control activities. Infection with the parasite can lead to severe skin disease, persistent itching progressing to visual impairment and blindness in early adulthood when people should be at their most productive. Onchocerciasis led to vast tracts of arable land being abandoned, reducing agricultural and economic productivity and exacerbating poverty in some of the poorest countries. Over 120 million people were at high risk, [5] an estimated 17.5 million people infected and 1.5 million suffered from visual impairment [3]. The socioeconomic importance of blindness due to onchocerciasis was the main reason for the first multi-partner, international control effort, the Onchocerciasis Control Programme (OCP)[6][7,8]. The OCP was established in 1974 initially in seven, and later covering 11 countries in West Africa. In the absence of a safe, effective drug, it was based on weekly aerial spraying with environmentally safe insecticides to control the blackfly vectors. Although the OCP was successful in reducing the transmission, incidence and impact of blinding onchocerciasis in large areas of the 11 countries, the disease remained unchecked in other endemic countries in West, Central and Eastern Africa. These countries were not covered by the OCP, as aerial spraying – the only control option available at the time - was not considered technically feasible or cost-effective due to the forested terrain[3]. The registration of Mectizan®(ivermectin MSD) in 1987, and the decision by Merck & Co. Inc. to donate the drug “ for as long as needed” for the treatment of onchocerciasis, radically changed control strategies[9][10]. Community trials confirmed ivermectin to be a safe microfilaricide, a single annual dose being effective for the reduction of the microfilarial load[11-13]. In 1988, the OCP introduced large-scale ivermectin treatment to supplement aerial spraying[2], initially using the mobile strategy with health workers responsible for treating the eligible members of the endemic communities. However, increasingly the communities themselves became involved in the programme management as the benefits of a community-based approach, piloted by the supporting NGOs, began to appear. In parallel, from 1992–1994 studies on the importance of skin disease and a cost benefit analysis were undertaken which provided scientific evidence for the need to expand the control of onchocerciasis to countries in Sub Saharan Africa outside the ambit of OCP [6,14,15].

In 1995, a second and much larger programme, the African programme for Onchocerciasis Control (APOC), was established to extend mass distribution of ivermectin to the other 19 endemic countries in Africa. The APOC programme is based on a broad international and national partnership focused on supporting CDTI. Sustained high coverage of annual treatments with ivermectin is necessary, making long-term commitment important. The programme now treats over 90 million people annually in 19 countries, protecting an at risk population of 115 million, and preventing over 40,000 cases of blindness every year [16].

After ten years of CDTI a dramatic reduction in prevalence and microfilaria was reported in sentinel villages of West province, Cameroon and five districts in Uganda[17], whilst after 15–17 years of ivermectin treatment in two onchocerciasis foci in Kaduna State, Nigeria, prevalence had fallen to zero level in all communities and all individuals examined were skin-snip negative[18] .

Although this global partnership involving WHO, the World Bank, national Ministries of Health, bilateral and multilateral donors, NGOs and Merck & Co. Inc. has been well documented [4,19-22], the critical role that NGOs have played in developing an approach to mass treatment undertaken by the affected communities themselves, has not been as well described apart from a recent review by Bush and Hopkins [4].We would like to explore this role in view of the current interest in community directed health interventions.

In Latin America, the Onchocerciais Elimination Programme of the Americas (OEPA) is also a partnership involving NGOs, but uses a different strategy of twice yearly treatment with ivermectin, but is not covered in this review [23].

### Role of NGOs in community based programmes and in health research for development

The World Bank defines NGOs as “private organizations that pursue activities to relieve suffering, promote the interests of the poor, protect the environment, provide basic social services, or undertake community development”. By definition then, NGOs are action–oriented and contribute to health and development of communities world-wide. Moreover, NGOs can encourage international donors to focus on health priorities of the country as well as good governance and accountability through their advocacy role [24] .

Lavis *et al *[25] defined research as “a knowledge loop- from generation of knowledge to its effective use”. In this “knowledge loop” NGOs are valuable partners in research for development where research is seen as a broad process, involving not only production of knowledge, but also downstream and upstream activities needed for relevance and effectiveness in setting priorities and translating knowledge into action [24]. NGOs can, and do, contribute to all different stages of health research – advocacy, priority setting, capacity building, resource mobilization, sharing and utilizing research findings and networking. NGOs are traditionally involved in activities which address health issues in resource–poor settings, often “at the end of the road” where national health services may not exist. Some NGOs undertake innovative field-based research - where the effectiveness of these innovations is often learnt by trial and error - and while these innovations may enhance effective and efficient implementation in the field, results are seldom analysed well or rigorously [24] .

The fact that NGOs are close to communities means they can play a critical role in interpreting evidence and translating it into relevance for those communities. NGOs are often involved in piloting new strategies and their subsequent scale-up. However, as NGO research is often conducted on a small scale and is usually qualitative in nature it often goes unrecognized by research organizations and funding agencies. The policy impact from experiences and lessons learnt by NGOs can be enhanced by partnerships with other key stakeholders.

### NGO involvement in onchocerciasis treatment programmes

The onchocerciasis story is a leading example of the contribution of a group of NGDOs^a^ to operational research which led to important changes in treatment strategies and policies.

The four case studies (below) illustrate some of the key contributions the NGDOs made to the development of what became “community directed treatment with ivermectin” (CDTI)- in particular the implementation and scaling –up of ivermectin distribution programmes. In onchocerciasis- endemic areas, health centres are generally few, in distant locations from communities and resource-poor in both commodities and qualified health personnel. The NGDOs provided resources to complement national programmes by supporting health care staff in remote communities.

From 1989 a group of (primarily) prevention of blindness NGOs independently worked with the health services to initiate mass-distribution of ivermectin and pioneer community–based strategies, initially in Mali and Nigeria. Although some key stakeholders were apprehensive about putting ivermectin into the hands of community volunteers, these early programmes received critical support of the OCP Director, Dr E.M. Samba. The improvement in coverage and compliance led to the eventual adoption by the OCP and the increasing acceptance of the importance of devolving authority to the community.

**Figure 1:**
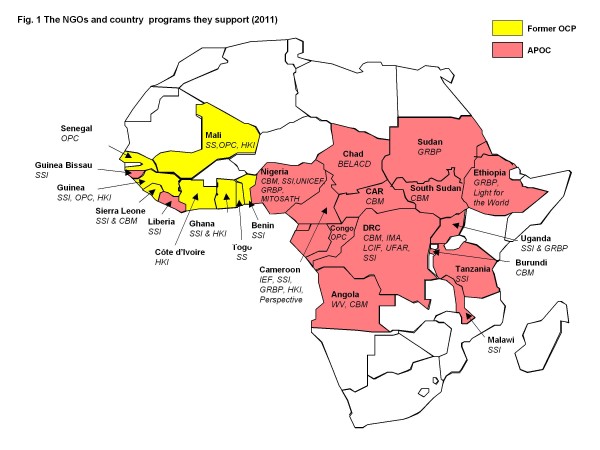
The NGDOs supporting ivermectin distribution programmes in Africa ( 2011).

The River Blindness Foundation (RBF), founded in 1990 specifically to broaden distribution of ivermectin [26], funded many of the early treatment programmes supported by individual NGDOs in countries outside of the OCP area. It soon became apparent that collaboration between the NGDOs would increase the effectiveness and efficiency of the treatment programmes and the NGDO Coordination Group for Onchocerciasis Control was created in 1991.

(Figure 1.) This group has met regularly ever since, and all those interviewed for the purpose of this document agreed that the collaboration has been a positive and constructive experience, which has not only supported the onchocerciasis programmes for 20 years but has also led to other joint initiatives in health. The NGDOs shared their innovations, experiences and lessons learnt in order to standardize methodology and increase effectiveness. The NGDO coordination group also worked collaboratively on approaches to donors for advocacy and fund-raising [4,10]. As WHO, Tropical Diseases Research (TDR ) and the Mectizan Donation Program (MDP) are included in the biannual meetings, important operation research issues are also addressed. One example was the need for a proxy for weight determination of ivermectin dosage. The early programmes had to weigh each person to determine the number of tablets they should take - which was not practical for mass treatment or a community-based strategy. The Nigerian National Eye Centre in Kaduna and research associates in London, NGDO personnel, and national programme staff collected the data on height /weight and optimal dosing categories [27]. This data allowed the Mectizan Expert Committee to endorse the height/weight treatment strategy in 1993, a change in policy which opened the door to a community–based approach to ivermectin distribution. Locally made colour-coded measuring poles simplified the correct dosing of each individual. These are now used by many other preventative mass chemotherapy programmes.

Collaboration between NGDOs was also needed at country level. In order to move away from a rather fragmented approach to support for ivermectin distribution, the NGDO coordination group encouraged the establishment of an NGDO coalition in each country. Building on the experiences of developing community-based approaches to onchocerciasis control pioneered by NGDOs and their government partners, TDR initiated multi-country studies to provide the kind of scientific evidence necessary for donors and policy-makers [28]. The TDR multi-country studies showed the potential of what became known as Community-Directed Treatment with Ivermectin (CDTI), as well as illustrating the value of NGDO partnership with WHO and TDR. CDTI was formally adopted by APOC in 1997 as its principal strategy. While their work contributed significantly to the development of CDTI, the scaling up of this new strategy was spearheaded by the NGDOs whose strength lies in working together with communities and governments of endemic countries.

**Figure 2:**
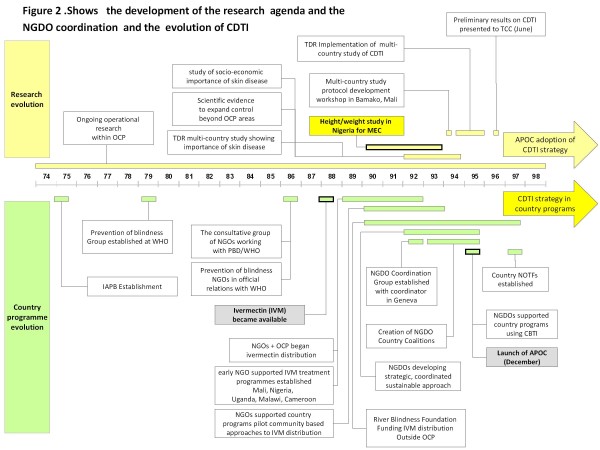
Milestones in the research and evolution of CDTI.

Figure 2 highlights key milestones in the development of the research cycle which led to improvements in programme implementation.

### Development of Community Directed Treatment with Ivermectin (CDTI)

There was a spectrum of activities that eventually led to CDTI, from a top down, ‘paramilitary’ approach of distribution of the ivermectin tablets by a mobile team of health workers, through the evolution of community-based treatment with ivermectin (CBTI), to the grass-roots Community Directed Treatment with Ivermectin (CDTI).

**Figure 3:**
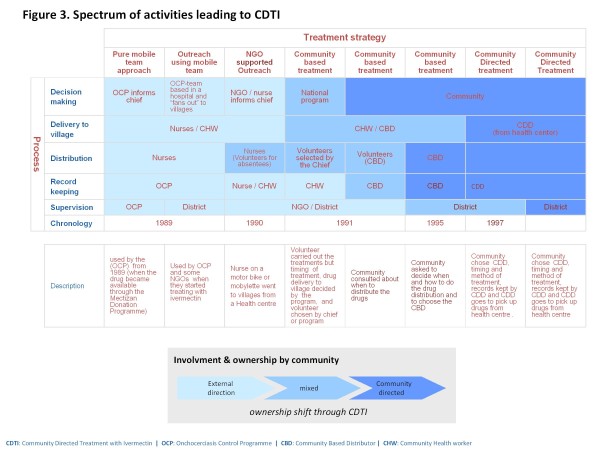
The spectrum of activities leading to CDTI.

These are illustrated in Figure 3. A simple and succinct explanation of the essential difference between Community–Based Treatment with Ivermectin( CBTI) and CDTI is: in CBTI the community is involved but is led; in CDTI the community is involved and leads the process, planning and management of treatment.

#### Case studies

in each of the APOC countries, the starting point for onchocerciasis distribution programmes was different. The following case studies from four countries that initiated early ivermectin distribution programmes supported by NGDOs illustrate some of the unique challenges they addressed.

## Case study 1: Mali

### Introduction

In West African savannah areas the prevalence of onchocerciasis was as high as 80–100 per cent by the age of 20 [29]. Further, virtually every person 40–50 years of age in hyper-endemic communities was either severely visually impaired or totally blind, and regions were frequently depopulated because of the high rates of blindness. This was the situation in Mali prior to onchocerciasis control activities. The area of Mali east of Bamako endemic for onchocerciasis was in the original Onchocerciasis Control Programme (OCP) area. The Western extension [1] later included the remaining areas of Mali west of Bamako. Larviciding operations of OCP certainly reduced the transmission of onchocerciasis significantly but impacted only slowly on infection and blindness due to disease. In 1987, after 12 years of vector control, a study to estimate the number of people infected and blinded by the disease, in order to prioritize populations for ivermectin treatment, showed communities in endemic foci were still at risk of onchocercal blindness [30]. The registration of ivermectin (Mectizan®) for the treatment of onchocerciasis in 1987 meant that the OCP could modify its strategy. In the original OCP area, ivermectin treatment was combined with vector control in areas where vector control had not been very effective. This combination strategy was used in some extension areas whilst in most parts ivermectin treatment alone was used.

### Early treatment strategies

The OCP started ivermectin treatments in Mali with a mobile team approach - this reflected the military–like operations that the OCP used with the aerial spraying operations. ‘*You do what you know how to –‘(*former NGDO Coordinator). At the beginning, the OCP headquarters in Ouagadougou decided on the timing of drug distribution, and provided the drugs and the vehicles. Furthermore, the EPI (Extended Programme of Immunization) team from Ouagadougou would go to the field with the national team to inform the village chief, and nurses would carry out treatment and monitor any side effects. OCP provided perdiems to both the OCP and national teams, which meant motivation for this mobile team approach was high, but not sustainable.

### Early NGDO involvement

In the early 1990s Sightsavers had begun to support eye care in a small way in Mali. In 1989, Sightsavers and CBM met to discuss the potential of ivermectin for prevention of blindness and Sightsavers decided to take the lead in Mali by assisting the OCP and the Ministry of Health in their aim to eliminate onchocerciasis as a disease of public health importance. Sightsavers appointed a River Blindness Coordinator in 1990 with the goal of promoting and supporting onchocerciasis control and integrating ivermectin distribution with other aspects of eye care in the countries where Sightsavers was present. In Mali, Sightsavers collaborated closely with the OCP in planning and logistics as all the drugs came though the OCP. In 1992 the Organisation Pour la Prévention de la Cécité (OPC) started working in the Kankan region of Guinea and expanded its ivermectin treatment programmes to Western areas of Mali, in collaboration with Sightsavers.

### The Pilot Programme

The River Blindness Coordinator joined Sightsavers after three years of working on phase iv trials of ivermectin in Liberia. He was already convinced of the safety of the drug and that a simple system to deal with adverse side effects could be set up without the need for a medical team. From 1991, Sightsavers pioneered community–based distribution which enabled treatment of a large number of people with the assistance of the community being treated. The community-based approach was piloted in the Baguineda sub-district of the district of Kati (the Koulikoro Region of southern Mali), in collaboration with the national onchocerciasis control programme, the District Medical Officer and sub-district health staff.

In the early community-based distribution approach, a community health worker (CHW) from the health centre (usually extension workers for immunization or TB) would go to the community, explain the programme and agree the best time for distribution. Community members would assist the CHW on the day of distribution. As the community-based approach evolved, community members achieved more “authority” and involvement, such as deciding when and how distribution would take place. The early Malian programmes did not use the dose poles, but age proxies : small children (under 5 s – determined by not being able to touch the opposite ear by reaching over their head) got nothing…. Up to 4 tablets for ‘fat/heavy/big people’.

When this method proved to be effective and acceptable - and following the 1991 meeting in Geneva on strategies for ivermectin distribution through primary health care systems - Sightsavers expanded its intervention to other districts. As the programmes expanded in 1993, Sightsavers and OPC coordinated with the OCP on which areas each NGDO would support.

The Baguineda programme was close to Bamako and was thus used by Sightsavers as a demonstration area for other programmes (e.g. Nigeria, Cameroon, Guinea Conakry) to come and learn how the programme operated.

### Challenges

At the outset this method of community participation met with some resistance from members of the Mectizan Expert Committee (MEC), the advisory committee on the use of the drug. They voiced concerns about the appropriateness of using volunteers and hence drug safety. The OCP senior staff also doubted whether solid data could be recorded for programme evaluation. Sightsavers had to demonstrate that ivermectin could be safely distributed with minimal supervision from health staff. The support of the OCP Director was very important for the NGDOs as he was able to interact at Ministry level and remove obstacles if there were problems with the national team.

*‘ We are on the verge of something very exciting ’* Sightsavers Director, after his visit to Mali, September 1991.

### Development of Community Directed Treatment with Ivermectin (CDTI)

As the community-based concept got established, and with the anticipated closure of OCP in 2002 and the “devolution” of onchocerciasis control to national teams in OCP countries, the need for installing a sustainable CDTI approach was critical. OCP recognised that this would enable the countries, at a minimum cost, to ensure that onchocerciasis would never remain a problem of public health importance after the end of OCP, thus fulfilling the objectives of the programme. However the change to community management and away from mobile teams initially met with considerable resistance from the national onchocerciasis control personnel who saw the loss of their per diem incentives.

CDTI really started after the protocol development workshop for the multi-country study on effectiveness and sustainability held in Bamako in 1994, when the concept of “community self-treatment” (later changed to Community Directed Treatment) was developed. Funding for the multi-country study was from the OCP, TDR and APOC. The Kayes region was part of the Malian component of this multi-country study.

### From Control to Elimination of Infection and Transmission

At the closure of the OCP in 2002, after 27 years of vector control and 12 – 15 years of ivermectin distribution, over 1.56 million people in the programme area were receiving annual treatment. These strategies were successful in interrupting transmission where vector control was effectively applied and succeeded in eliminating onchocerciasis as a public health problem [2]. Longitudinal studies started in 2005 in Mali and Senegal to determine whether the parasite could be eliminated through ivermectin treatment only. The first results have provided empirical evidence that elimination of onchocerciasis with ivermectin treatment is feasible in some endemic foci in Africa [31], and the principle of elimination has since then been established.

### Integrating onchocerciasis programmes with other health and development activities

In Mali, the original goal of Sightsavers was to integrate the onchocerciasis treatment programmes with primary eye care, but this was not possible until the CDTI was established and strong, although early work took place to provide rehabilitation services for people blinded by the disease. By 2005, the onchocerciasis control programmes had been integrated into the comprehensive eye care in the Sightsavers and OPC supported projects. They supported the training of the Community Directed Distributors (CDD) in primary eye care, cataract and trachoma referral. Helen Keller International (HKI) promoted the delivery of vitamin A to infants - a simple intervention that meant that even those not eligible for ivermectin received an important health benefit. The integration between the onchocerciasis and lymphatic filariasis (LF) programmes at the country level helped to demonstrate the economic benefit of an integrated approach to disease control/elimination. The Neglected Tropical Diseases programme is now based entirely on the CDTI strategy .

*‘Learn to listen to the community - When Dr. Samba visited Mali, it was the villagers who said to him “ Give us the drugs and we will distribute them. You must treat everyone in the village, even those on the other side of the River”’.* (Former OCP Director).

## Case Study 2 : Nigeria

### Introduction

Onchocerciasis, first reported in northern Nigeria in 1908 [32], was widespread throughout the country. Ocular onchocerciasis, the second leading cause of blindness in Nigeria, was prevalent in parts of northern Nigeria, [33] and onchocercal skin disease (OSD) was widespread in the rain forest and savanna/mosaic areas[34,35].

In 1956 Budden estimated the number of infected persons at 339,000, with about 20,000 blind due to onchocerciasis[32]. However, following the comprehensive nationwide prevalence survey by the national onchocerciasis control programme (NOCP) from 1987 to 1990, it was realized that the disease was prevalent in all but three of its 36 States and that nearly 30 million people needed treatment. Nigeria accounts for nearly 40% of the world’s burden of onchocerciasis[36].

Vector control measures were initiated in the early 60s in certain river basins and the WHO later opened an onchocerciasis research centre in Nigeria. The NOCP with its Technical Advisory Committee was established in 1982, and in 1987 task forces were established at national, zonal and State levels, and State Onchocerciasis Control Units were formed in the endemic States. However, the control of onchocerciasis in Nigeria posed a major challenge to the government.

The adoption of a federal structure after independence in 1960 meant that health services including onchocerciasis control activities were provided concurrently by the Federal and State governments, and later by the local government areas (LGAs) after their creation in 1976. In 1988, following the Alma Ata declaration, Nigeria adopted a primary health care (PHC) policy and strategy for universal coverage, with the local government level as the central fulcrum for control activities [37], supported by the State and Federal governments.

### Introduction of ivermectin in Nigeria and early involvement of NGOs

In 1989, when Merck’s donation of Mectizan®for the treatment of human onchocerciasis was announced, the International Eye Foundation (IEF) and Africare managed to get a two year grant from the Public Welfare Foundation to support the Ministry of Health in distribution of the drug ivermectin in Kwara State (now part of Kogi State). Ivermectin distribution in Kaduna State was also initiated in 1989 with support from Sightsavers.

### Mobile treatment: challenges

The system of distribution by mobile teams of health workers faced many challenges including coverage, both therapeutic and geographic. The substantial cost of implementation to the NGDOs and health system, the reluctance to take the drug by the people in some affected communities, and the reluctance of health workers to work in areas where ivermectin was needed, were major obstacles to be confronted. Treatment coverage was low because the working hours of health personnel coincided with the time people were on their farms, or had other community activities. The health workers did not spend enough time in the villages for communities to understand the purpose of their visit, recognise the potential benefits of ivermectin and the need for long-term use of the drug. ‘*I recall a visit to a village in Kaduna State where the village was totally empty due to a funeral’* (former National Eye Centre Director)*. ‘Many communities associated the treatment with birth control’ (*Carter Center Country Director).

For their part, as elsewhere, policy makers were apprehensive of possible adverse effects of ivermectin and its safety in large scale community use. The correct application of the exclusion criteria was of major concern, especially as experience during the community trials revealed that individuals concealed pregnancies and illnesses in order to benefit from deworming effects of the drug.

In parallel with Sightsavers’ work in Mali, the IEF/Africare supported projects in Kwara State were also piloting a community-based approach. As the former Africare Country Director explained, in an interview in 2011, ‘*There were a lot of restrictions on the administration of ivermectin, but no clear guidelines so we felt we had to violate the rules in order to get started’.* In 1991*,* after the presentation of Africare and Sightsavers on the feasibility of community-based treatment with ivermectin (CBTI) at the WHO in Geneva and to NOCP Nigeria, the decision was reached to move towards the CBTI strategy.

Early in 1993, the NOCP Zone C Coordinator for onchocerciasis accepted an invitation by Sightsavers to visit the pilot CBTI programme in Mali. In the report he submitted on his return he stated that ‘*Above all, the community based distribution which entails maximum participation by the communities themselves appears to hold promise for the success and sustainability of the Mectizan distribution programme’.* This strategy, which was found to be less expensive, ensured that, once trained, community members, as opposed to health workers, provided the treatments themselves. The mobile treatment was progressively replaced by the community-based treatment strategy.

By 1996 CBTI expanded to all endemic States, supported by a number of international NGDOs and a local NGDO, MITOSATH. It was agreed to assign a single NGDO per State. All endemic States with the exception of Akwa Ibom were therefore provided with NGDO or UNICEF support, and an NGDO Coalition was formed the same year to work with the NOCP. UNICEF was also instrumental in helping with the logistics of ivermectin supply and, together with the NGDOs, helped build accountability into the program.

Although there were little in the way of side effects, there were limitations of the CBTI strategy, as noted by the Carter Center Country Director, ‘*Community-based distributors were appointed by the community leaders not selected by community members. The CBDs were over worked because it was only one CBD per community. CBDs’ accessibility and acceptability due to political/clannish differences were major challenges’*.

### The development of Community-Directed Treatment with Ivermectin (CDTI) in Nigeria

With the creation of APOC, the CBTI approach was found not to be the best solution to building sustainable distribution of ivermectin. The communities needed to take ownership of the programme, as ivermectin distribution might well be required for at least 20 years if transmission of infection was to be interrupted. *‘While CBTI implied that the treatment programme is placed within the community, the health workers, health systems and NGDOs still played a major role.’*(UNICEF Onchocerciasis Coordinator). ‘*CDTI on the other hand meant that the communities took ownership of the programme and decided on how to run it after training by the health workers and NGO partners ’* (former National Eye Centre Director)*.*

In 1997, the NOCP in partnership with the NGDO Coalition adopted the CDTI strategy with support from APOC. The CDTI strategy transferred most of the responsibility for onchocerciasis control to the affected communities and this strategy revolutionized the programme in Nigeria. It led to a massive increase in the number of health staff and community personnel trained and treatments dramatically increased from about 6 million in 1996 to 27.4 million by 2010. By 2008, about 98% of target communities were being covered and a minimum of 75% therapeutic coverage had been sustained for over 5 years. Over 30,000 communities, many of them in remote areas, have been reached.

In the expansion of the CDTI strategy to all endemic areas, the UNICEF Coordinator noted ‘*From headquarter to States, the NGOs built the capacity of both the health workers and the communities to deliver ivermectin; developed the guidelines and made it available across the country’.* At the same time, he recognized the facilitating roles of APOC and the Nigerian government. The secretariat of APOC in Ouagadougou led the process of launching approved CDTI projects in Nigeria and other countries in the programme, but the catalytic role of the NGDOs was important: *‘The NGOs were a catalyst that propelled the implementation of CDTI in the country’* (former CBM Country Director ).

By 1998, ivermectin treatment in parts of south-east and south-west Nigeria was reported to have led to a reduction in some of the clinical symptoms of onchocerciasis [34]. Recent epidemiological surveys supported by APOC have shown that onchocerciasis transmission may have been interrupted in Zamfara, Kaduna, Ebonyi, Enugu, parts of Cross Rivers and Taraba States following 12 to 16 years of Ivermectin treatment[16].

The partnership between the Ministry of Health and NGDO Coalition in Nigeria has brought about completion of mapping of onchocerciasis, increased and sustained treatment coverage, initiation and expansion of other community-based interventions, as well as the provision of a huge personnel resource base, particularly at the community level, contributing to strengthening health systems. It also facilitated the submission of proposals to and access of funds from APOC and assisted with the reporting on those funds.

### Development and scale-up of other interventions in Nigeria using CDTI as a vehicle

Having made significant progress in the delivery of ivermectin using the CDTI approach, the NGDOs began including other health interventions which communities needed. One example is the distribution of albendazole for the elimination of lymphatic filariasis (LF) and praziquantel for the control of schistosomiasis in Plateau and Nasarawa States by The Carter Center, beginning in 1999. Between 2001 and 2009, Sightsavers, HKI, and CBM began to include in the existing onchocerciasis treatment programmes Vitamin A supplementation, primary eye care, rehabilitation of the blind and distribution of insecticide-treated nets for malaria control. The supporting NGDOs took the lead in ensuring appropriate training of health staff, as well as the availability of health education materials and drugs or equipment. In some cases, communities themselves started asking their CDDs experienced in onchocerciasis control to be become distributors of additional interventions.

Challenges in co-implementation and integration were encountered - *‘With LF integration to onchocerciasis control there was no reaction but when we began to integrate other programmes such as trachoma and malaria we started to experience some resistance to integration for fear of the unknown, resistance to change, scepticism and the issue of territorial turfs’*.(Carter Center Country Director**).** However once resistance was overcome, the benefits of joint training, implementation and monitoring were appreciated, as was the cost benefit.

This partnership, founded on the onchocerciasis programme, paved the way for the emergence of a coordinating mechanism for Neglected Tropical Diseases (NTDs) at the Federal level, and has contributed to the development of an NTD plan of action for the country. As the UNICEF Coordinator observed there is still more potential ‘*CDTI should be linked to programmes supported by NGDOs that can benefit hugely from the CDD network, for instance, the Maternal & Newborn week. If the CDTI concept could be brought into the Maternal and Newborn week programmes- that would be novel and successful’.*

## Case study 3 : Cameroon

### Introduction

Before control activities, onchocerciasis was endemic in much of the country, and indeed some of the seminal work on the existence of different strains of *Onchocerca volvulus*, savanna and forest onchocerciasis, and *O.volvulus-Simulium* complexes was carried out in Cameroon[38][39]. Severe ocular lesions and blindness occurred in the Touboro region (Sudan savanna regions) of north eastern Cameroon with up to 90% prevalence [40], whilst 95% prevalence and high microfilarial loads, but limited ocular involvement, were reported from forest areas of Cameroon (Central, South and Southwest provinces).

In the 1980s and 90s, the Edea region, Vina valley in the north and the Sanaga valley in South province were areas of intense research programmes on the epidemiology, transmissions and mass treatment with ivermectin (carried out by Tubingen University and ORSTOM/ IRD^a^ in collaboration with the University of Yaoundé). These programmes involved routine skin snipping and blood sampling which was to have a negative impact on the community acceptance of mass treatment.

Cameroon, along with Nigeria, was one of the first countries to complete the rapid epidemiological mapping of onchocerciasis (REMO)[41,42] which delineated the main foci and determined the at risk population to be 3.5 million - 50% of the total rural Cameroonian population [42]. The REMO and REA (rapid epidemiological assessment) were critical for the success of the APOC programme and the National Onchocerciasis Coordinator at the Ministry of Public Health ( MoPH) in Cameroon played an important role in the development and implementation of the REMO.

### Early NGDO involvement and introduction of ivermectin

In 1991 the River Blindness Foundation (RBF) was funding the International Eye Foundation (IEF), and later Helen Keller International (HKI), who were carrying out pilot treatment programmes in Southern Cameroon and the Sanaga river basin. In 1992 RBF started its own treatment programme in Garoua (northern Cameroon). CBM supported hospital based programmes at Acha Tugi, Ngaoundéré and Enongal. At the same time, research programmes using Mectizan were supported by TDR and GTZ (German aid) in the Vina valley and Edea regions.

The early ivermectin distribution strategy was a mobile team/outreach approach, similar to that used for other interventions e.g immunization. The NGDOs trained nurses from the health centres who would go to the villages to distribute the ivermectin, having sent information beforehand to announce their arrival. However coverage and compliance were a problem and it was seen early on that the timing of drug distribution was not right and the community needed to be involved in the discussions and decisions regarding this. The then RBF Country Director said *“In some communities the turnout was very poor so we went back and asked why?”* This was echoed by the former HKI Onchocerciasis Director *“The communities explained that it was the harvest/rains/not a convenient time so then HKI consulted with the community on timing of distribution.”*

After informal discussion of these issues, the NGDOs involved increasingly moved to a community-based approach to distribution in line with practice in other endemic countries. In 1994, the NGDO coalition was formally established with IEF, HKI, RBF, and the Lions Clubs (Sightsavers joined in 1995).

### Challenges

The ivermectin distribution programmes in Cameroon faced several difficult challenges, as there were two issues that set Cameroon apart from many of the other APOC countries.

1. Cameroon had adopted the Bamako initiative, and onchocerciasis control efforts had to be integrated into the local primary health care system (PHC) which included a cost recovery component.

2. Safety - the problems associated with ivermectin treatment of people co-infected with *Loa loa* and *O.volvulus* (See Co-infection with Loa Loa).

In terms of the first challenge, MoPH insisted on using the cost recovery mechanism which caused tension with the NGDO Coalition. The Ministry was also concerned about the handling of the drugs by village health workers with only basic training, let alone by untrained community members. In addition, nurses at the health centres who benefited from the cost-recovery system were reluctant to relinquish their role. On the part of the communities there was also reluctance: *‘You had to convince the men that it was worth giving up the equivalent of one beer to treat his family’* (former HKI Country Director). ‘*The fact that people had to pay for a drug that would prevent them from going blind or getting skin disease was a real problem when they did not feel sick’* (Former RBF Director).

Sightsavers’ country representative reported in 1996 that ‘*The Ministry is still clearly concerned about sustainability and proper integration with the PHC programme – but the door was described as no longer closed to a CBD approach – it couldn’t be described as wide open either’.*

*‘ The project in Cameroon will certainly be a challenging one …. The challenge for Sightsavers is to establish an effective distribution system in the project area so that the Ministry can be convinced of the merits of a community-based distribution system’ (*Sightsavers’ Regional Director and River Blindness Programme Coordinator).

### Transition to CDTI

By 1996 the MoPH had accepted the principle of involving the community in the management of the programme but on condition that community members did not handle the drugs. This was, in part, due to a previous bad experience where some community health volunteers, who had been trained in basic PHC activities, went beyond their mandate, setting themselves up as “little doctors”. This programme was scrapped - just before the CBTI strategy was introduced.

Cameroon launched its first APOC projects in 1998/9,however the combination of cost recovery, the use of the limited number of health staff available to undertake distribution, and reported side effects resulted in Cameroon reporting the lowest (therapeutic and geographic) coverage. It was eventually acknowledged by the Ministry that cost recovery was a significant barrier to uptake of the drug by community members and the decision was made that ivermectin would be free. The NGDOs and other APOC partners played an important role in persuading Cameroon to adopt the CDTI strategy and from then on coverage improved, although the Government decision to pay the CDDs meant that there were often interruptions in the distribution when payment was late.

### Co-infection with Loa Loa

Safety was the biggest challenge for both the NGDOs and the MoPH in Cameroon. Shortly after the first mass treatment programmes began in central and southern Cameroon, serious neurological adverse events were reported in patients with very high *Loa loa* microfilaraemia [43] . This had a major impact on programme implementation in terms of timing, roll-out, supervision, training and monitoring. Epidemiological surveys were undertaken to determine the area co-endemic for loasis and onchocerciasis and at-risk populations. Monitoring procedures were established and adhered to during distribution so that people developing serious reactions would receive prompt, appropriate treatment [44,45]. The NGDOs worked with the national programme to identify the health staff that were available to supervise treatments, timetable the process village by village, ensure that the drugs to treat SAEs were available (often funding them), and that health staff knew what to do, where to refer and how to report in the event of an SAE. A rapid assessment tool (RAPLOA) was developed by TDR for assessing prevalence of *L .loa* as it was determined that the risk of severe adverse reactions was unacceptable in onchocerciasis-endemic communities where more than 20% of the population also has loiasis [46]. A strong communication strategy was devised by APOC and the MoPH. This, combined with effective case detection and management, and enhanced treatment supervision in which the NGDOs played a major role, allowed the CDTI programmes to continue, although any patients being treated for the first time are considered to be at risk.

Despite these challenges the CDTI is now a flagship programme for Cameroon and the Government is proud of the CDD achievements – some of whom have been involved in the programme for more than 15 years and are now involved in other interventions. With APOC and NGDO support, Cameroon has successfully integrated several other activities into the CDTI, including home malaria treatment, bed net distribution, vitamin A and other mass chemotherapy ( Mectizan + albendazole, mebendazole).

*‘ When you put confidence in the community, they are capable of managing the project. CDTI has served as a channel for health interventions to improve access to services and better health’* (former National Onchocerciasis Coordinator).

## Case study 4: Uganda

### Introduction

In contrast to the policy debates which took place in Cameroon, Uganda embraced community involvement in ivermectin distribution relatively easily.

There were, however, significant differences between West African countries and Uganda in terms of the disease itself – the nature of the predominant vector - and the health service structure. In Uganda, onchocerciasis is mainly meso-endemic and is confined to foci in the west and northwest of the country with a small focus on the border with Kenya around Mount Elgon. The disease is transmitted by two vectors *Simulium damnosum s.l.* (common in West Africa) and *S. neavei.* Described by Fischer et al in 1993[47], the latter accounts for 70% of transmission in Uganda. The fly has a short flight range which makes it less able to transmit the disease to neighbouring areas by comparison with *S. damnosum* with its much longer flight range.

In the early 1990s it was estimated that over 2 million people in 5000 communities were at risk from onchocerciasis and in 1993 a national control programme was launched. From 1997 the programme benefited from the support of the recently established APOC. The affected communities were actively involved in managing the ivermectin distribution from the beginning, as the health services were increasingly decentralised, and based at village level on the so-called Resistance Councils, established in the late 80s after President Museveni took power. The Resistance Councils and their role are described below in the section on The Pilot Programme.

### Early NGDO involvement

As early as May 1988, Sir John Wilson recommended to the Royal Commonwealth Society for the Blind (RCSB) their intervention in onchocerciasis control in Uganda, following a meeting with a former Minister of Health who reported re-emergence of the disease which had been subject to vector control in the 1940s and 50s. In 1989 an Ophthalmic Clinical Officer based in Fort Portal, studied onchocerciasis as part of his post-course attachment after attending a Community Eye Health course in London. In his report of November 1989 to the RCSB (now Sightsavers), he claimed that the disease was ‘*a widespread problem in Western Uganda’*, citing particularly Kabarole, Kasese, Bushenyi, Hoima and Rukungiri Districts.

The following year an Advisory Committee to the Ministry of Health on Onchocerciasis was set up. This comprised Ministry staff as well as representatives of NGOs, including Sightsavers, the Uganda Foundation for the Blind and the Church of Uganda. A meeting in February 1990 was called to discuss a proposal for a pilot programme of distribution of ivermectin tablets in Hoima and Masindi districts, which Sightsavers agreed to support.

### The pilot programme

The seriousness of the disease and the need for action in Hoima and Masindi are highlighted in the November 1990 report of the Sightsavers River Blindness Coordinator’s visit. He visited the two districts during the initial distribution of ivermectin, met with health staff and community members, and noted widespread itching of the skin. Some people were said to be thinking of abandoning the area because of the fly nuisance and the effects of the disease, despite the fertility of the soil. There were local reports that *‘there were many people blinded by the disease’* and, in the area in Masindi thought to be hyper-endemic, ophthalmologists saw people with onchocercal eye lesions. The mass treatment programme in Hoima and Masindi began in earnest in 1991, with support from Sightsavers and its partner, the Uganda Foundation for the Blind.

The report of the February 1990 meeting spoke of the need to ‘*train and use a general Medical Officer and his local staff to do mass distribution of ivermectin and monitor, treat and report side effects of the drug’.* Although use of health staff to undertake distribution was initially the agreed policy in Uganda, the local Resistance Council was relied on from the beginning to assist with the census to determine who needed treatment. The Resistance Councils quickly assumed a critical role in relation to ivermectin distribution, as described by the Masindi DMO, in a letter to Sightsavers on 27 February 1991. It is worth quoting at length.

"*‘Uganda happens to have a Unique Community Organisation system where the grass-root Unit is the Resistance Council comprising a village or two depending on the population density, For purposes of Community based health care, we have adopted the same grass root unit to divide the population into the various communities. Each of these communities, besides having a Resistance Committee of nine people also has a Health Committee of seven people…*"

"*The distribution team members and the Community leaders sit and discuss the issue. The latter are charged with the responsibility of under-taking household registration… At the time of the Ivermectin distribution, the health team convenes at the Community selected centre and the distribution is done household by household as organised by the Community leaders. The distribution team members now record the activities in a distribution register indicating the name, sex, age and weight of Ivermectin recipient. The dose given is recorded and space left to record any side effects. For the household members who are not eligible, the contra-indication is noted and space left for subsequent action.*"

"*This community based distribution method ensures comprehensive coverage and continuity. It also serves to involve the Communities who are now able to supervise the members of the distribution team. Furthermore, it paves the way for other health intervention activities and therefore in general serves to improve the health of the people.’*"

So by 1991 community based methodology was already evolving in Uganda and its use for other health activities was foreseen.

### Development of community-based methodology

Reliance on health teams to undertake the actual distribution quickly revealed its limitations in terms of treatment on a mass scale. Reports from 1991 speak of stretched resources, high cost per treatment because of the allowances paid to the personnel involved, and above all low coverage. Sightsavers’ River Blindness Coordinator recalls that in a re-training seminar in Hoima in January 1992, ‘*It was agreed to increase supervision and to simplify the distribution method, using the “Mali” model: village volunteers had to be actively involved in the actual distribution of ivermectin, while the MOH personnel would function as “field supervisors”’.* The 1992 Annual Onchocerciasis Report for Masindi and Hoima Districts described the role of the distributor as follows:

‘On voluntary basis the distributor has the following duties to perform:

· *He/she has to teach and advise his/her people in the village about onchocerciasis and the use of ivermectin tablets*

· *He/she has to keep the tablets which are given to him/her by the supervisor/field officer for distribution to his/her people in the village*

· *He/she must register all, thus every person and household in his/her village and keeps the records in the register*

· *Distributes ivermectin tablets to all the eligible people*

· *Observes and reports the complications due to ivermectin tablets to supervisors/field officers.’*

The National Onchocerciasis Coordinator recalls that initially health workers were not happy with the changes, and especially with communities taking responsibility for the drugs, but that ‘*it did not take long to change their opinion and see that community members could handle both drugs and side effects’*. By 1994, with the communities now undertaking community-based treatment, coverage was improving.

### Steps towards a national programme

In July 1991 a delegation from the River Blindness Foundation of the USA visited Uganda to discuss the development of a national plan to combat onchocerciasis with the MOH and partners. This was preceded by a visit to Sightsavers in the UK to discuss potential collaboration. The RBF established the Global 2000 River Blindness Programme which supported the Ministry of Health to establish a countrywide control programme which began in 1996. RBF provided funds until APOC began support in 1997. At that point The Carter Center took over RBF activities and effectively became the lead NGDO co-financing the control of onchocerciasis in Uganda, while Sightsavers continued to support the original districts, Hoima and Masindi (which later became three when Hoima was divided in two, Hoima and Kibaale). By then CDTI had become the methodology for ivermectin distribution accepted by all partners within APOC.

### Integrating onchocerciasis programmes with other health and development activities

In 2001 a meeting took place in Entebbe, funded by APOC, to review the use of the community-directed approach to initiate other health activities. Following on from this, the governing board of APOC asked TDR to undertake a study on CDI. The TDR research on *Community-directed interventions for major health problems in Africa*[28] included a study site in Uganda (Arua, Sironko, Kyenjojo, Kanungu and Nebbi districts). This multi-country study looked at the use of CDTI methodology pioneered in the onchocerciasis programmes to address other health interventions, namely Vitamin A distribution, insecticide-treated bed nets and home-management of malaria, and directly observed treatment for TB (DOTS), managed alongside ivermectin treatment. The conclusion of the three-year study in 2008 was that ‘*where already established for onchocerciasis control, the community-directed intervention (CDI) approach should be used for the integrated, community-level delivery of appropriate health interventions’*.

In Hoima, Masindi and Kibaale. Sightsavers and the district health authorities used the working relationship built up through ivermectin distribution to address eye disease generally. By its nature as a blinding disease, the onchocerciasis programme identified many people with visual problems and some who had lost their sight altogether. In 2000 it was agreed to develop Comprehensive Eye Services (CES) in the three districts. CES is an internationally recognised model for eye care - a continuum of prevention of blindness (principally onchocerciasis and trachoma control, cataract surgery and provision of spectacles for those who need them), rehabilitation services for people already blind, and educational support to blind children in mainstream schools. A critical factor is the willingness of sectors within government and supporting NGOs to build a referral system so that, for instance, a child with low vision is provided with the spectacles and other devices and the support to enable him/her to progress in school, or that an adult diagnosed blind from onchocerciasis is referred to rehabilitation services. An evaluation of CES in the three districts in 2004 found that despite some shortcomings – for example, transfer of trained personnel, over-reliance on NGO funding – a referral system for people with eye problems was in place and working and that relations between the government organs and the voluntary sector were strong.

The success of the network, from community to national level, established through the onchocerciasis programme in the 1990s, was a factor which led to Uganda becoming one of the first countries to develop an integrated package for the control of Neglected Tropical Diseases.

## Conclusions

In summary, these case studies illustrate how the community-based approach was piloted by an NGDO in Mali and was implemented on a much larger scale in Nigeria. Although both countries eventually embraced CDTI, challenges of training, supervision, logistics and accountability needed to be addressed by the NGDOs and national programmes. In Cameroon, the NGDOs and APOC had to convince the government to change policy and adopt CDTI as well as facing the major challenge of dealing with the Loa-related SAEs. In contrast, the Ugandan health system was already open to community involvement, quickly embraced CDTI and has now officially adopted CDTI for other health interventions. Understanding the local health and social environment, combined with a gradual and flexible approach which demonstrates the benefit of community involvement are the main lessons that emerged.

The success and advantage of CDTI for onchocerciasis control over other community models comes from its ability to mobilise communities to take on the responsibility for an activity of a short duration (part-time over a few weeks) once a year. The packages of drugs are not too large and can be easily transported on the back of a bicycle; calculation of dosage is easy with the height poles; the exclusion criteria are not too difficult to determine; and the system of recording usage can be handled by people without high levels of literacy. CDTI is thus suitable for unpaid village volunteers. It does not take them away from their other responsibilities for too long, and the community can find ways of compensating them such as by helping them with their farm work or building a house. Compare that to the difficulty of transporting and delivering heavy insecticide-treated bed nets, or monitoring the combination of drugs necessary for TB patients over a period of months (DOTS).

In addition to their role in programme development the NGDOs also contributed to research on height/weight data to allow use of height for mass treatment in Nigeria and its subsequent uptake; ensured collaboration and coordination on treatment strategies and policies, coalition building, and advocated and raised funds for onchocerciasis control at national and international level. The resources which NGDOs brought to national programmes helped with a range of activities including briefing new health officials on programme implementation, troubleshooting where treatment was not going well and generally helping the national staff to manage the programmes better.

Moreover, NGDOs initiated the integration of other appropriate health interventions into CDTI, starting with HKI and the inclusion of Vitamin A supplementation. Although integration is generally lauded as a positive development – the challenges are also recognized. At what point does a CDD, or others in the village willing to volunteer their time, become overloaded to the detriment of standards and increasing community fatigue? As the former TDR disease Coordinator, said *‘The question is – will the community–based approach really be sustainable once the onchocerciasis programmes scale back or is it the driving force?’*

For Merck and the Mectizan Donation Program the active participation of non-governmental development organizations has been particularly important to the continuing success of the MDP. In the early years of the programme, NGDOs working in the area of blindness prevention throughout Africa served as a catalyst for the delivery of ivermectin. Then NGDOs provided substantial funding and are still prepared to raise funds to provide support to these long-term programmes. They continue to play a critical role, particularly in countries where local health services lack appropriate and necessary resources [9]. As a result of 25 years of the Merck Mectizan Donation programme, the OCP and APOC partnerships more than 800 million dose of ivermectin have been given to over 90 million people[48]. Onchocerciasis has been significantly reduced in more than 25 countries and focal transmission likely to be interrupted in at least 10 countries[48]. Recent estimates in the decline of infection, severe itch, visual impairment and blindness for 15 APOC countries from 1995 to 2010 show that control operations have averted more than 6·3 million DALYs, at a relatively nominal cost of US$ 257 million[49]. With annual doses for 10–19 years, ivermectin, renamed by rural communities as a “wonder drug”, has dramatically lowered the prevalence of onchocerciasis[16]. Recent publications on the feasibility of elimination of onchocerciasis in Africa[18,31] with ivermectin alone, underscored the effectiveness of the CDTI strategy and the power of this unique partnership .

As Cupp *et al *[48] state, *‘Ivermectin may have changed the face of tropical medicine more than any other drug this century’*. The introduction of ivermectin enabled the evolution of new, effective and sustainable strategies of community-based health interventions which are now being adopted for other diseases. Indeed, the neglected tropical disease (NTD) agenda would not have been feasible without the CDTI framework.

The transformation of outreach treatment strategies to community- based methodologies was a natural evolution. However, there is general consensus from the various stakeholders and partners interviewed that the transition to community-based treatment and then to CDTI would not have happened without NGDO involvement - or if it did it would have been very slow. NGDOs were the driving force- their strength being in outreach and community engagement. ‘*NGDOs played a key role and will continue to play a key role when the APOC finishes- they will still be there’* (former APOC Director).

As stated earlier, it is widely acknowledged that the success of control of onchocerciasis in Africa was due to the partnership between governments of endemic countries, the international donor community, NGDOs, international health and development experts, research institutions, and the effectiveness of the control strategies. While OCP and APOC provided leadership, the NGDOs working with the national health authorities played a major role in developing the community methodology which led to achieving the treatment of 90 million people with ivermectin each year, and which was further developed for use by other health and development programmes.

The NGDOs have played an evolving role over the last 15 years, working increasingly closely with the WHO on policy, political commitment and advocacy – with a greater clarity of their role and purpose.

## End note

^a^ In 1993 the NGO coordination group for ivermectin distribution made the decision to use the term Non-Governmental Development Organization, NGDO to emphasize the development focus of their activities

## Abbreviations

APOC, African Programme for Onchocerciasis Control; BELACD, Bureau d’Etudes de Liaison des Actions Caritative et de Développement; CBD, Community based distributor; CBTI, Community based treatment with ivermectin; CDD, Community directed distributor; CDTI, Community directed treatment with ivermectin; CHW, Community health worker; EPI, Expanded programme on immunization; TCC, The Carter Center; HKI, Helen Keller International; IEF, International Eye Foundation; IAPB, International Agency for the prevention of blindness; IMA, IMA World Health; IVM, Ivermectin; MEC, Mectizan expert committee; MDP, Mectizan Donation Program; MoPH, Ministry of Public Health; NGO, Non-governmental organization; NGDO, Non-governmental development organization; NOCP, National onchocerciasis task force; NOTF, National onchocerciasis task force; OCP, Onchocerciasis control programme; OPC, Organisation pour la prévention de cécité; ORSTOM, Organisation de la recherche scientifique et Technique Outremer. It has since been replaced by IRD (Institut pour la Recherche et le Developpement); RAPLOA, Rapid assessment procedure of loasis; RBF, River blindness foundation; TB, Tuberculosis; TCC, Technical consultative committee; TDR, UNDP/World BANK/WHO Special programme for research and training in tropical diseases; WHO, World health organisation.

## Competing interests

The authors declare they have no competing interests.

## Author’s contributions

SEOM & UVA conducted interviews with the key stakeholders and designed the outline of the paper. SEOM wrote the first draft, UVA & CC provided input and comments. SEOM, UVA and CC drafted one or more of the case studies. All authors read and approved the manuscript.
